# Civilisation and the Deterioration in Physique

**Published:** 1907-03-30

**Authors:** 


### CIVILISATION AND THE DETERIORATION IN PHYSIQUE.

In the midst of our advancing civilisation there is a latent distrust of civilisation itself. We are never weary of proclaiming the enormous gain it has brought to manners, to morals, and to intellect; but there is a widespread impression that the benefit is purchaesd by a corresponding physical decay. Fortunately for the hopes of man the alarm is unfounded. The advance of accurate knowledge dispels it. Civilisation is cultivation of the whole nature of man; and even in its present imperfect state it not only permits physical training, but promotes it. The traditional glory of the savage body is under medical statistics fading away. It is becoming evident that the average barbarian, observed from the cradle to the grave, does not know enough and is not rich enough to keep his body in its highest condition. On the contrary, the savage is small, sickly, short-lived, and weak, compared with the man of civilisation. The great athletes of the world have been civilised; the most long-lived men have been civilised; the most powerful armies have been civilised; and the average of life, health, size, and strength is highest to-day among those races where knowledge and wealth and comfort are most widely spread. However far back we go, admiring memory looks further. Homer and Virgil remind us that their modern heroes only live in glass houses, to have stones thrown at them. Lucretius and Juvenal chant the same lament. Xenophon, mourning the march of luxury among the Persians, says that modern effeminacy has reached such a pitch that men have even devised coverings, called gloves, for their fingers.

Herodotus narrates that, when Cambyses sent ambassadors to the Macrobians, they were asked what the Persians had to eat and how long they commonly lived. The reply was that they sometimes attained the age of eighty, and that they ate a mass of crushed grain which they termed bread. On this, the inquirers said it was no wonder that the Persians died young when they partook of such rubbish, and that probably they would not survive even so long but for the wine they drank; while the Macrobians lived on flesh and milk, and survived 120 years.

But, these facts apart, there are certain general truths which look ominous for the reputation of the *physique* of savage tribes. First, they cannot keep the race alive, they are always tending to decay. When first encountered by civilisation, they usually tell stories of their own decline in numbers, and after that the downward movement is accelerated. They are poor, ignorant, improvident, oppressed by others' violence, or exhausted by their own; war kills them, infanticide cuts them off, pestilences sweep them away, and whole tribes perish by famine and small-pox. Both under the stern climate of the Esquimaux and the soft skies of Tahiti, the same decline is seen. Parkman estimates that in 1763 the whole number of Indians east of the Mississippi was but 10,000, and they were already mourning their own decay. Travellers seldom visit a savage country without remarking on the scarcity of aged people and of young children. Lewis and Clarke, Mackenzie, and Alexander Henry observed this among Indian tribes never before visited by white men. Civilisation keeps alive, in every generation, multitudes who would otherwise die prematurely. These millions of invalids do not owe to civilisation their diseases, but their lives. It is now satisfactorily demonstrated that the picked survivors of savage life are commonly suffering under the same diseases as their civilised compeers, and show less vital power to resist them. The earliest missionaries to the South-Sea Islands found ulcers and dropsy and hump-backs there before them. The English Bishop of New Zealand, landing on a lone islet where no ship had ever touched, found the whole population prostrate with influenza. Lewis and Clarke, the first explorers of the Rocky Mountains, found Indian warriors ill with fever, dystentery, rheumatism and paralysis; and Indian women in hysterics. "The toothache," said Roger Williams, of the New England tribes, "is the only paine which will force their stoute hearts to cry"; even the Indian women, he says, never cry as he has heard "some of their men in this paine"; but Lewis and Clarke found whole tribes who had abolished in the civilised manner this source of tears by having no teeth left. We complain of our weak eyes as a result of civilised habits, and Tennyson, in "Locksley Hall," wishes his children bred in some savage land, "not with blinded eyesight poring over miserable books." But savage life seems more injurious to the organs of vision than even the type of a cheap edition; for the most vigorous barbarians often suffer more from ophthalmia than from any other disease; without knowing the alphabet they have worse eyes than if they were professors, and have not even the melancholy consolation of spectacles. Most formidable of all is the absence of all recuperative power in the savage who rejects civilisation. No effort of will improves his condition; he sees his race dying out, and he can only drink and forget it. But the civilised man has an immense capacity for self-restoration; he can make mistakes and correct them, sin and repent, sink and rise. Instinct can only prevent; science can cure in one generation and prevent in the next.

In the robust days of Queen Bess the teeth of the Court ladies were habitually so black and decayed that foreigners used constantly to ask if the English ate nothing but sugar. Hurtzner, who visited this country in 1697, speaks of the same calamity as common among all classes.

The armour of the knights of the Middle Ages is too small for their modern descendants. Hamilton Smith records that two Englishmen of average dimensions found no suit large enough to fit either of them in the great collection of Sir Samuel Meyrick. The head of the Oriental sabre will not admit the English hand, nor the bracelet of the Kaffir warrior the English arm. The swords found in Roman tumuli have handles inconveniently small; and the great mediæval two-handed sword is now supposed to have been used only for one or two blows at the first onset, and then exchanged for a smaller one. The statements made by Homer, Aristotle, and Vitruvius, represent six feet as a high standard for full-grown men; and the irrefutable evidence of the ancient doorways, bedsteads, and tombs, proves the average size of the race certainly not to have diminished in modern days.

The enormous savage races have turned out, as has been shown, to be travellers' tales, even the Patagonians being brought down to an average of 5 ft. 10 in., and being, moreover, only a part of a race, the Abipones, of which the other families are still smaller. Indeed, we can all learn, by our own experience, how irresistible is the tendency of the imagination to attribute vast proportions to all hardy and warlike tribes. Most persons fancy the Scottish Highlanders, for instance, to have been a race of giants, yet Charles Edward was said to be taller than any man in his Highland army, and his height was but 5 ft. 9 in.

The most careful investigations give the same results in respect to physical strength. Early travellers among the Indians, as Hearne and Mackenzie, and early missionaries to the South-Sea Islands, as Ellis, report athletic contests in which the natives could not equal the better-fed, betterclothed, better-trained Europeans. When the French savants, Péron, Reguier, and Rausonnet, carried their dynamo-metres to the islands of the Indian Ocean, they found with surprise that an average English sailor was 42 per cent. stronger, and an average Frenchman 30 per cent. stronger, than the strongest members of the island tribes they visited. Even in comparing different European races it is undeniable that bodily strength goes with the highest civilisation. It is recorded in Robert Stephenson's Life that when the English navvies were employed upon the Paris and Boulogne railway, they used spades and barrows just twice the size of those employed by their Continental rivals, and were regularly paid a double wage.

From accurate statistics we know that the duration of life in every nation is a fair index of its progress in civilisation. Quetelet gives statistics, more or less reliable, from every nation of Northern Europe, showing a gain of 20 to 25 per cent. during a half-century. Where the tables are most carefully prepared the result is least equivocal. Thus in Geneva, where accurate registers have been kept for 300 years, it seems that from 1560 to 1600 the average lifetime of the citizens was twenty-one years and two months; in the next century, twenty-five years and nine months; in the century following, thirty-two years and nine months; and in the year 1833, forty years and five months: thus nearly doubling the average age of man in Geneva during three centuries of social progress. In France it is estimated that, in spite of revolutions and Napoleons, human life has been gaining for nearly a century. In a manuscript of the fourteenth century, moreover, it is shown that the rate of mortality in Paris was then 1 in 16—one person dying annually to every sixteen of the inhabitants. It is now 1 in 32—a gain of 100 per cent. in 500 years. In England the progress has been far more rapid. The rate of mortality in 1690 was 1 in 33; in 1780 it was 1 in 40; and it stands now at 1 in 60—the healthiest condition in Europe; while in half-barbarous Russia the rate of mortality is 1 in 27. It would be easy to multiply these statistics to any extent, but they all point one way, and no medical statistician now pretends to oppose the dictum of Hufeland that "a certain degree of culture is physically necessary for man and promotes duration of life." The simple result is that the civilised man is physically superior to the barbarian.

Domestication is not weakness. A strong hand does not become less muscular under a kid glove, and a man who is a hero in a red shirt, will also be a hero in a white one. Civilisation, imperfect as it is, has already procured for us better food, better air, and better behaviour; it gives us physical training on system; and its mental training, by refining the nervous organisation, makes the same quantity of muscular power go much farther.

